# Erythrocytes Nanoparticle Delivery: A Boon for Targeting Tumor

**DOI:** 10.34172/apb.2023.080

**Published:** 2023-04-29

**Authors:** Adnan Rehmatullah Siddique, Geeta Sameer Bhagwat

**Affiliations:** ^1^Pharmaceutics Department, HK College of Pharmacy Mumbai 400102, India.; ^2^Pharmaceutics Department, DY Patil University School of Pharmacy, Sector-7, Nerul, Navi Mumbai 400706, India.

**Keywords:** Erythrocytes nanoparticles, Biomimetic nanoparticles, Cancer therapy, RBCs, Targeted delivery

## Abstract

Although nanoparticles (NPs) have many advantages as drug delivery systems, their poor stability in circulation, premature drug release, and nonspecific uptake in non-target organs have prompted biomimetic approaches to camouflage nano vehicles using natural cell membranes. Among them, which are extensively studied in erythrocytes, are the most abundant circulating blood cells. They are specially used for biomimetic coating on artificial NPs due to their excellent properties of good biocompatibility, biodegradability, non-immunogenicity, and long-term blood circulation. Erythrocyte-mimicking nanoparticles (EM-NPs) are prepared by combining nanoparticle cores with naturally derived erythrocyte (red blood cell or RBC) membranes. Compared with conventional nanosystems, EM-NPs hold the preferable characteristics of prolonged blood circulation time and immune evasion. In this review, the biomimetic platform of erythrocyte membrane-coated NPs is described in various aspects, with particular focus placed on the coating mechanism, preparation methods, characterization method, and recent advances in the biomedical applications of EM-NPs concerning cancer and targeted delivery.

## Introduction

 Administration of drugs through the systemic route is the most widely used approach for delivering a drug to the targeted area for treating several indications and diseases; however, administration of drugs through the systemic route suffers from some drawbacks, such as some drugs’ poor absorption and bioavailability, a drug’s low therapeutic index, or the development of multiple drug resistance, severe side effects, and non-specific targeting. All such drawbacks can be overcome by developing a drug delivery system like nanoparticles (NPs). A nanoparticle is a type of drug delivery in which the drug is encapsulated within a protective core, and these NPs transport API to the desired targeted sites of action with reduced toxicity and side effects. Nanomedicine has emerged as a thriving research topic over the last two decades, with a wide range of products developed, including nanocarriers, nanovaccines, nanophotensitizers, and nanoprobes, some of which have already been approved by the Food and Drug Administration (FDA).^1^

 Despite recent significant advances and promising results in nanomedicine, NPs-based drug delivery has encountered a number of difficulties and setbacks. These challenges and setbacks include

 Biological and compatibility issues,

 Batches are scaled up from small to large scale.

 Biocompatibility, stability, and safety,

 Different regulatory aspects for different regulatory bodies

 All of these issues with NP-based drug delivery make clinical translation to market a costly and time-consuming process.

 Till now, we learned in general about the nanoparticle drug delivery system and what the challenges were. Now we can see the problem associated with non-biomimetic NPs. First and foremost, immune detection and clearance by mononuclear phagocytic cells are issues that NPs face, limiting their therapeutic use.^2^ Several scientists attempted to overcome this limitation in various ways, one of which was marked by the addition of polyethylene glycol (PEG) functionalization to the surface of the NPs. By avoiding immune evasion by the reticuloendothelial system and mononuclear phagocytic system, this functionalization enhances long-term circulation in the body.^3,4^ However, on repeated administration of PEG-modified NPs, they lose their efficacy by activating the immune response.^5^ Additionally, only those NPs that manage to get beyond the biological barriers stated above can interact with the target tissue or organ; it was recently found that only a small portion of the NVs administered reach solid tumors.^6^ To circumvent the constraints of nonbiomimetic NPs, a new biomimetic cell-membrane-based technique was developed that mimics various natural mechanisms to produce the desired effects. This approach has been researched to develop stealth and targeted NVs.^7^ The invention of long-term circulation NPs and their specialised targeting of certain tissues and organs were inspired by different cell membranes from leukocytes, platelets, erythrocytes (red blood cells, or RBCs), cancer cells, and microorganisms.^8^ Among them, erythrocytes are the most widely studied because of their remarkable drug delivery properties, such as their long blood circulation (up to one twenty days in humans), which makes these cells one of the most suitable carriers for the delivery of a variety of therapeutic active compounds like proteins, drugs, and enzymes; apart from that, the mature RBC lack a nucleus and other organelles, making the process of membrane extraction and purification more simple.^9,10^ After removing the internal components of RBC via hypotonic lysis or another method, the membranes recovered are used to coat NPs using extrusion, sonication, or microfluidic electroporation.^11^ The use of RBC membranes for coating NPs is done mostly because of the inherent biocompatibility and biological qualities of their parent cells, which aid in the retention of their surface proteins, allowing them to function normally. Notably, erythrocyte-mimicking (EM)-NPs have been extensively studied for a variety of applications such as drug administration, imaging, phototherapy, nanovaccines, and nanoantidotes,^12^ implying a high potential for treatment conversion and a significant influence in a variety of treatments. As a result, in this article, we present an overview of recent advancements in the biological uses of EM-NPs in the context of cancer.

## Method of preparation for Erythrocytes mimicking nanoparticles

 There are various methods available for encapsulating the bioactive drug into the erythrocyte membrane, which are based on physical and chemical properties such as:

 Hypotonic hemolysis^13,14^

 Hypotonic dilution^15,16^

 Hypotonic dialysis^17^

 Hypotonic preswelling^18,19^

 Osmotic pulse^20^

 Chemical perturbation of the membrane^21,22^ along with electrical breakdown^23^

 Different molecules are encapsulated through endocytosis, lipid fusion, and the intrinsic uptake of substances by erythrocytes.^24^

 To prevent leaks from the loaded erythrocytes, which could result in toxicological problems, and to obtain proper stability, the encapsulated compounds should have a significant degree of water solubility as well as not react with the erythrocytes’ membrane, i.e., they should not form any physiochemical interactions with erythrocyte membranes.

###  Preparation of erythrocyte-mimicking derived vesicles (EMVs)

 The preparation of EM-NPs is generally divided into two parts: the first is to obtain membrane-derived vesicles from RBCs, and the second is to incorporate NPs into membrane-derived vesicles by vesicle-particle fusion.^18,22,23,25^

 Thus, EM-NPs are made by combining two steps: hypotonic treatment to get ghost RBCs devoid of internal components, followed by sequential extrusion of these particles and membrane to produce NPs shrouded in RBC membrane.

####  General procedure

 Fresh blood is obtained from the organism (for example, a mouse) and centrifuged at 4°C to preserve protein activity before the upper layer comprising platelets is discarded to collect RBCs.

 Obtained RBCs are washed with phosphate-buffered saline (PBS) multiple times and re-collected by centrifugation to remove any residual plasma and other unwanted cells.

 Ghost RBCs are obtained by hypotonic treatment, which involves gently mixing washed RBCs with an excess of 0.25 PBS and holding them to release the intracellular RBC constituents.^25^

 After centrifugation to remove hemoglobin, the ghost RBCs in the pink precipitate are sonicated and pushed through various pore sizes of polycarbonate membrane pores with the use of an Avanti mini-extruder to get the desired RBC vesicle size.

 Protease inhibitors are normally added to the samples and they are refrigerated at 4°C to keep the membrane bioactive.^26–28^

**Figure 1:**
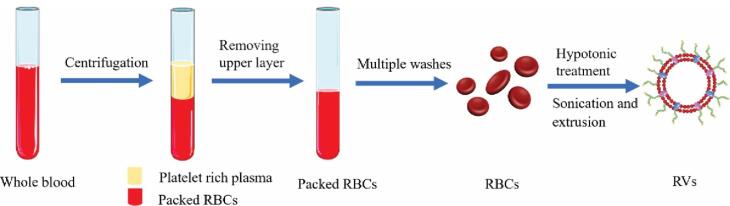
Schematic presentation of the preparation of the ghost RBC vesicle; Adapted from the open access article under the CC BY-NC-ND license (http://creativecommons.org/licenses/by-nc-nd/4.0/).^11^

 These RBC vesicles are further used for coating the NPs, utilising various vesicle particle fusion techniques. Whole processes are explained in Figure 1^11^ in pictorial form.

## How do EMVs form a coat around the NPs?

 RBCs have a flexible structure that is dependent on viscoelasticity, cell surface-to-volume ratio, and the cell content viscosity of the cell membrane, allowing them to move through restricted capillary networks and “sieving organs” like the liver and spleen with ease. The glycocalyx, a rich polysaccharide covering the surface of RBCs, is critical for cell stability and immune evasion.^29,30^ For spatial stability, these complicated polysaccharides on the cell surface are akin to a hydrophilic coating.^31,32^ The stabilised EMV’s surface can effectively limit further membrane contacts, but polymeric NPs with higher surface energies are most likely to interact with the stabilised membranes of the polysaccharide to decrease total energy.^33^ In the presence of high concentrations of EMVs, this stabilising process ensures the formation of a monolayer film coating. Furthermore, the negatively charged sialyl groups in the polysaccharide terminus confer charged asymmetries on the cell membranes, which is important for EMV-nanoparticle interfacial interactions. Luk et al,^33^ found that negatively charged NPs could create nuclei-shells with separate particles, but positively charged NPs only formed polydisperse aggregates. The presence of a densely negatively charged sialic acid moiety on the outer membrane side increases the possibility of such outcomes. When strongly positively charged NPs with a high affinity for negatively charged sialic acid combine, the lipid bilayer of the membrane is likely to collapse, preventing the local arrangement necessary for lipid coverage. In contrast, the electrostatic repulsion between the sialic acid moiety and the negatively charged NPs allowed the NPs to merge with the intracellular membrane side, establishing a right-side-out membrane orientation structure to preserve cell surface glycocalyx. Thus, it should be kept in mind during the preparation of EM-NPs that the charge on the EMVs and the NPs should be the same to obtain stable EM-NPs.

## Methods of vesicle-particle fusion

 The first attempts at interconnecting NPs and EM-NPs used “bottom-up” techniques, in which NPs were functionalized through RBC surface chemistry. Moreover, the use of chemistry-based bioconjugation techniques for preparing RBC-mimicking delivery vehicles resulted in protein denaturation. Hu et al ^34^ described a “top-down” method for creating camouflaged NPs from erythrocytes in 2011. They effectively wrapped the sub-100-nm PLGA NPs with the erythrocytes by extruding NPs with nanoscale EMVs produced in advance. This “top-down” strategy is among the most promising approaches for large-scale EM-NP manufacturing. Several major erythrocyte nanoparticle fusion methods are briefly summarised here.

###  Co-extrusion method 

**Figure 2:**
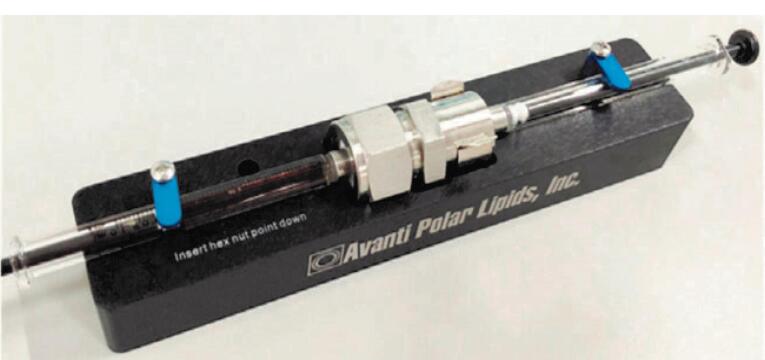
Co-extrusion method; Adapted from the open access article under the CC BY-NC-ND license (http://creativecommons.org/licenses/by-nc-nd/4.0/).^35^

 In this approach, the manufactured NPs are commonly fused with acquired EMVs using mechanical extrusion, which makes use of a mechanical extruder. The interfacial interactions between both the NPs and the EMVs, which were previously mentioned, constitute the principle involved in this coating process. The produced NPs and EMVs are repeatedly injected through porous membranes of various sizes numerous times before being sonicated for several minutes, depending on the size required^33^ (Figure 2).^35^ This approach coats NPs by providing sufficient energy for the vesicle-particle collision during extrusion. To reduce membrane protein loss and degradation, the EM-NP composed of phospholipids should be as complete as feasible during the preparation procedure.^34^ Excessive empty vesicles result from successive extrusions; these surplus vesicles are separated by centrifugation, with the precipitate representing the finished product and redispersed for further use.^25^ RBC membrane volume and the total membrane volume necessary to thoroughly encapsulate 1 mg of NPs are used to calculate the ratio of EMVs to NPs.^33,34^ To determine the effect of nanoparticle size on the required number of EMVs, Luk et al^33^ used PLGA NPs with diameters ranging from 65 to 340 nm and covered them with erythrocyte membranes, resulting in varying amounts of EMVs required. The number of EMVs required for a given weight of NPs is determined by their size; the smaller the particle size, the fewer RBC membranes are required. It should be noted that during the extrusion process, cell membranes are lost. To compensate for this loss during preparation and the fusing process with NPs, large volumes of EMVs are commonly used to ensure that all nanomaterials are covered with EMVs.

###  Microfluidic electroporation methods 

**Figure 3:**
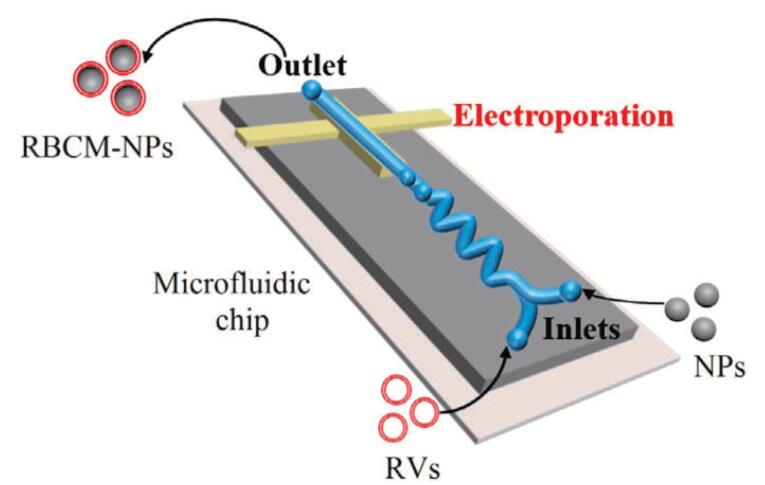
Microﬂuidic electroporation method; Adapted with permission from Rao et al.^36^ (Copyright © 2017 American Chemical Society).

 As the usage of biomimetic NPs grows in popularity in the biomedical field, one technology that has shown promise in the production of EM-NPs is microfluidic electroporation. The use of this technique was demonstrated by Rao et al,^35^ who used the microfluidic device to coat the Fe3O4 magnetic nanoparticles (MNs) with EMVs. The device contains a chip, which is a microfluidic chip that is used for electroporation (Figure 3).^35^ The device is divided into five sections:

- Two inlets for EMVs and NPs
- Merging channels in the shape of a Y and mixing channels in the shape of an S.
- Zone of electroporation
- Outlet.

 When a mixture of NPs and EMVs passes through the electrokinetic zone, they are exposed to electrical pulses created in the electroporation zone. These electrical pulses deliver enough energy to cause the dielectric layer on biological membranes to disintegrate, resulting in many temporary pores,^36^ which will allow NPs to enter the RBC membrane. The most important element to consider during these processes is that the pulse voltage, timing, intensity, and rate of flow should all be correctly controlled and optimized. The EM-NP is obtained from the outlet after unification. The microfluidic electroporation method is the best technique because it perfectly combines biology with physics. It also has an advantage over the co-extrusion method in that constantly squeezing NPs through membrane pores does not necessitate a lot of force, and EM-NPs prepared by this technique have better membrane integrity compared to those prepared by the co-extrusion method. Furthermore, EM-NPs made using microfluidic electroporation had greater colloidal stability and in vivo efficacy than those made using traditional extrusion methods. As a result, the use of microfluidic electroporation for the creation of bioinspired NPs seems to have a promising future.

###  Cell membrane-templated polymerization 

**Figure 4:**
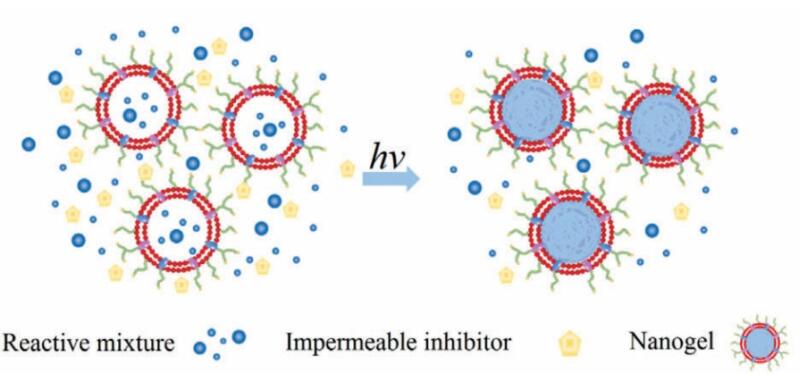
Cell membrane-templated polymerization. Adapted with permission from Zhang et al.^38^ (Copyright © 2015 Wiley Online Library).

 The majority of current methods for coating NPs with RBC membranes are based on nanoparticle template coating pathways, in which the nanoparticle centre is manufactured first and then the nanoparticle is covered with biomimetic membrane co-extrusion and microfluidic electroporation techniques. The problem associated with these techniques is that the interfacial interactions^33^ between both the RBC membranes and the nanoparticle cores, which may prevent the encapsulation of some non-compliant nanomaterials, are a problem with these approaches. This problem prompted us to consider the idea of nanoparticle cores being generated in situ in vesicles derived from cells. Such a possibility was explored by Zhang et al,^37^ who effectively executed the first example and successfully used a cell membrane-template polymerization approach to synthesise polymer cores by in situ polymerization to produce cell membrane-coated nanogels for the first time (Figure 4)^37^. They used acrylate polymerization as a model system, with the main goal of studying the effect of adding a membrane-impermeable complex molecular inhibitor during membrane-templated formation, which was created by combining a common membrane-permeable free radical scavenger, 2, 2, 6, 6-tetramethyl piperidine-1-yl-oxyl, with PEG. The macromolecular inhibitor’s main purpose is to effectively stop extracellular agglomeration while retaining the internal responsiveness of the vesicles, lowering the risk of cellular membrane denaturation from protein and content leakage.^37^ After adding the macromolecular inhibitor, UV irradiation was used to stimulate the gelation process, which resulted in the creation of cell surface-coated bioinspired nanogels. This approach has several advantages over coating nanoparticle templates, including thorough coverage of the nanocores and easy control of the final bioinspired nanoparticle size and stiffness. As a result, other than nanogels, the cellular membrane template polymerization technology is projected to become more acceptable for covering diverse nanostructures in the near future.

## In vitro characterization of EM-NPs

 In the following section, various parameters for in vitro characterization of these biomimetic NPs will be discussed, as it is necessary to assess these biomimetic NPs for their chemical structures and outer membrane proteins, as these factors play a major role in their immune evasion and long-term circulation.

 The following are some of the most essential characterization techniques for biomimetic NPs:

1. Size and surface morphology
2. Characterization of surface proteins
3. Fluorescence co-localization
4. UV–visible absorption spectra

###  Size and surface morphology 

 Dynamic light scattering measurements were used to compare the size of the NPs and their potential values before and after EM-NP encapsulation. Because the cell membrane lipid bilayer thickness is roughly 8 nm, the diameter of the coated NPs is commonly between 10 and 20 nm. After surface coating, the surface energy is close to the empty EMVs.^25,38,39^ Furthermore, when compared to naked NPs, EM-NPs have been shown to have a stabilising effect.^40^ EMVs, NPs, and EM-NP form and morphology are often observed using transmission electron microscope (TEM) and scanning electron microscopy (SEM). Original rough and irregular cell membrane fragments, when repeatedly extruded from the extruder into 100–200 nm hollow spherical vesicles, will give a micron-level concave disc with a regular cell membrane structure completely different from untreated RBC morphology when observed under electron microscopy. It was found that the hollow spherical vesicles provide sufficient space for a nanoparticle to be encapsulated. If there is a successful nanoparticle coating, it can be confirmed via negative staining and TEM. In negative-stained EM-NPs examined in TEM, a layer of membrane covering the nanoparticle surface is visible (i.e., the core-shell structure)^28,33,34,41^

###  Surface protein characterization

 The presence of certain erythrocyte proteins in the encapsulated NPs, which determine whether the wrapped NPs will have immunological escape and long-term circulation effects, is another sign of effective coating. The RBC, EMVs, and EM-NP proteins are seen by sodium dodecyl sulfate-polyacrylamide (SDS-PAGE) gel electrophoresis followed by Coomassie staining, with all protein bands having a profile identical to that of the RBCs.^28,41^ Western blotting analysis was used to further investigate specific protein markers. The presence of glycoprotein A,^42^ sialic acid glycoprotein, and the blood group A antigen was discovered in EMVs and EM-NPs, with CD47 found in roughly equal amounts on RBCs, EMVs, and EM-NPs.^43^ For a protein expressed on the EM-NP surface, the biological potential of CD47 to avoid macrophage phagocytosis can also be investigated at the cellular level. EM-NP absorption by mouse macrophage RAW264.7 cells, in particular, was reported to be 59.0% lower than that of bare NPs.^28^ Furthermore, the EMVs coating has reached saturation in CD47 functionalization, at which point about 92% of the supplied membrane proteins are utilised for particle functionalization, implying that the whole coating process is both plausible and achievable.^41^ Once EMV-specific proteins have been coated on NPs, it is extremely desirable to determine their orientation and position. Using cell blebs as an intermediary, signal molecule surveillance and moment scaling spectrum analysis were employed to investigate the relationship between membrane proteins and lipids.^44^ However, there is another approach to analysing this interaction that is based on the geometry that is developed by automatically inserting a known 3-dimensional structure of membrane protein into membranes.^45^ These approaches or techniques can be found useful in illuminating RBC protein orientation.

###  Colocalization of fluorescence 

 Hu and colleagues^34^ devised a method in which hydrophobic red DiD (1,1’-dioctadecyl-3,3,3’,3’-tetramethylindodicarbocyanine, 4-chlorobenzenesulfonate salt) colours and lipophilic green rhodamine-DMPE dyes were loaded into the nanoparticle polymer cores and EMVs before their formation to further analyse and confirm The dual-fluorophore-labeled NPs were then cultured with HeLa cells for 6 hours, following which a fluorescence microscope revealed that DiD and rhodamine DMPE overlapped at the same spot. After being swallowed by the cells, fluorescence colocalization revealed that the NPs had a perfect core-shell structure, showing the effectiveness of the EMV coating.

###  UV–visible absorption spectra 

 UV-visible spectroscopy is another technique that can be used to confirm whether the encapsulation of the nanoparticle is complete or not. In this technique, there is a comparison between the absorption peaks of the original nanoparticle and the encapsulated one. The NPs will give a specific absorption peak at a specific wavelength, but the encapsulated NPs will give two specific absorption peaks, one at the wavelength of the NPs, and the other will show a specific absorption peak similar to the EMV’s absorption peak.^25,46^ This indicates that the EMVs were successfully shifted onto the nanoparticle surface without compromising the original nanoparticle properties.

 All of these in vitro evaluation techniques, when combined, may simply confirm the optimal state of erythrocyte separation into vesicles and nanoparticle integration.

## Application

###  EM-NPs in cancer 

 With an expected 9.56 million deaths worldwide in 2018, cancer is one of the most life-threatening diseases.^47^ Chemotherapy’s effectiveness as a backbone in clinical tumour treatment is hampered by significant side effects, which are mostly due to low absorption, poor tumour selectivity, large dose requirements, nonspecific action, and multidrug resistance.^48^ This entire problem has encouraged the scientist to develop a drug delivery system that can be successfully used to improve

 the pharmaceutical properties of therapeutic molecules,

 the targeting of the drug directly to the cell or tissue,

 the therapeutic window by increasing efficacy, safety, and reducing toxicities and

 The simultaneous administration of various medications promotes therapeutic efficacy and combats drug resistance.^49,50^

 Furthermore, quick blood clearance, early drug release during blood circulation, unspecific targeting, and poor tumour penetration remain key roadblocks to the clinical translation of many nanomedicines. These restrictions led the scientist to develop bio-mimetic methods using natural cell membranes like RBC to camouflage the NPs, giving them the ability for immune evasion and to easily cross biological and physical barriers. Chemotherapy, phototherapy, radiation therapy, and immunotherapy have all benefited from the use of EM-NPs, which are currently being studied in preclinical trials.^11^

####  Drug delivery

 Several studies enlighten the potential benefit of EM-NPs as cancer drug delivery platforms for their ability to escape immune activation and prolong circulation in the blood. One of the first studies, for example, looked into several ways for loading doxorubicin (DOX), using polylactic acid (PLA) as a core nanoparticle and encapsulating it with an RBC membrane. The PLA@EM-NP findings revealed that chemical conjugation produced a more prolonged release of the drug than physical encapsulation and that the RBC membrane acts as a barrier, reducing drug diffusion by 1.2 times.^51^

 Using a lymphoma mouse model, Luk and colleagues showed that erythrocyte membrane-coated poly (lactic-co-glycolic acid) (PLGA) NPs (PLGA@EM-NP) loaded with DOX controlled tumour growth better than the free drug or non-coated NPs. Furthermore, as compared to free DOX, these EM-NPs virtually doubled the overall survival rates from 24 to four weeks for the control group and elicited a stronger immunological response.^52^

 Others created nanocrystals of paclitaxel (PTX) covered with RBCs (PTX-PEG@EM-NP). As a result of the cell membrane thickness, PTX-PEG@EM-NP particle size was slightly larger (327.5 ± 9.5 nm) than PEGylated NPs (295.53 ± 8.03 nm). Furthermore, the PTX-PEG@EM-NP zeta potential was 8.8 0.5 mV, which was equivalent to the zeta potential of the RBC membrane, indicating that the erythrocyte membrane concealment on the surface of the PEGylated NP was successful. In comparison to non-coated PTX-nanocrystals, when these PTX-PEG@EM-NP NPs were tested for anticancer effectiveness, they showed improved tumour accumulation and a nearly two-fold decrease in breast tumour development while also reducing significant side effects.^53^ In one study, EM-NPs were used to administer numerous chemotherapeutic drugs simultaneously to overcome tumour cell heterogeneity and treatment resistance. This research was carried out by Fu and colleagues, who evaluated different coverings for chitosan NPs, such as PEG or erythrocyte membranes, for the co-delivery of PTX and DOX. When compared, the bioinspired coated NPs have been found to have a two-fold increase in avoiding macrophage uptake, tumour cell retention, and cytotoxicity. When compared to the conventional PEG surface coating.^54^ Su et al and Chai et al conducted research to improve tumour targeting and tissue penetration. In this study, EM-NPs were combined with tumour cell-penetrating proteins like RGD, resulting in a 5-6-fold increase in therapeutic efficacy with increased tumour targeting and penetration.^28,55^ Su et al discovered that EM-NPs with a nucleus of poly (caprolactone) (PCL) loaded with PTX had a blood circulation time 5.8 times faster than parental polymeric NPs. Furthermore, when compared to non-coated NPs, PTX-loaded EM-NPs coupled with iRGD (arginine-glycine-aspartic) (EM-NPs/iRGD) had a 5.59-fold superior penetration ability and strongly inhibited over 90% of murine breast tumour growth and suppressed 95% of lung metastasis formation, making them much more effective than PTX-loaded EM-NVs or non-coated.^28^ Another study used docetaxel (DTX) nanocrystals coated with erythrocyte membrane, which contributed to high drug loading, long-term stability, and a nearly 6-fold increase in systemic retention time as compared to the free drug. The EM-NPs that were transformed with the tumor-targeting peptide (RGDyK) had much better tumour accumulation and therapeutic activity.^55^

 To envision new anticancer therapies, EM-NPs have also been studied for the delivery of natural substances with antitumor characteristics, like gambogic acid (GA) or curcumin.^56,57^ Zhang discovered that GA-loaded PLGA NPs coated with erythrocytes suppressed the proliferation of the human SW480 colorectal cancer (CRC) cell line. When compared to non-coated GA-PLGA NPs or free GA, this erythrocyte membrane-coated NPs method reduced CRC tumour development in vivo by nearly one-fold or three-fold and increased animal longevity.^58^ Other researchers used curcumin, a well-known naturally occurring anti-tumor compound. In this study, researchers encapsulated curcumin into porous PLGA NPs coated with RBC membranes and observed a larger cellular uptake of the coated NPs than the non-coated NPs by cancer cells while avoiding macrophage phagocytosis. Furthermore, when compared to non-coated counterpart NPs or free curcumin, RBC membrane-coated curcumin-loaded NPs inhibited tumour growth by triggering tumour cell apoptosis in a mouse H22 hepatocellular carcinoma xenograft model.^59^ These results indicated that EM-NP cloaked drug delivery systems could enhance natural compound anticancer effectiveness while reducing side effects. Table 1 summarises the use of EM-NPs to improve the delivery of therapeutic medicines for cancer treatment.

**Table 1:** EM-NPs to improve the delivery of therapeutic drugs for application in cancer

**EM-NVs composition**	**Drug used**	**Major advantage**	**Therapeutic** **efficacy**	**Experimental model**	**Ref**
PLA@EM-NP	Doxorubicin	Enhanced cytotoxicity and drug release profile	~2 fold**	In vitro, Kasumi-1 leukemia cell line	^51^
PLGA@EM-NP	Doxorubicin	Enhanced therapeutic efficacy, immunocompatibility, and safety	2 fold	Murine EL4 lymphoma	^52^
PTX PEG@EM-NP	Paclitaxel	Higher EPR, higher loading efficiency, and therapeutic efficacy	~1.5 fold	Murine 4T1 breast tumor	^53^
CMC-Fe3O4@ EM-NP -RGD	Doxorubicin + Paclitaxel	Toxicity is reduced, tumor retention is improved, and the therapeutic effect is enhanced.	~6 fold**	Murine LLC lung tumor	^54^
PCL@ EM-NP -iRGD	Paclitaxel	Increased tumor penetration, blood circulation time, and therapeutic response	5.6-Fold	Murine 4T1 breast tumor	^28^
DTX-PF127@ EM-NP -RGDyK	Docetaxel	Drug loading, tumor accumulation, and antitumor effectiveness are all enhanced.	3-Fold** 3-Fold***	U87 glioma xenograft	^55^
PLGA@ EM-NP	Gambogic acid	GA solubility and therapeutic effectiveness have both been enhanced.	~1-Fold ~3-Fold**	SW480 CRC xenograft	^58^
PLGA@ EM-NP	Curcumin	The uptake of tumor cells and treatment efficacy have both improved.	~2-Fold ~3-Fold*	H22 hepatic tumor xenograft	^59^

Carboxymethyl chitosan (CMC); Pluronic F127 (PF127); Poly (caprolactone) (PCL); Poly (ethylene glycol) (PEG); Poly (lactide acid) (PLA); Poly (lactic-co-glycolic acid) (PLGA). When compared to non-coated (or similar) NPs, therapeutic effectiveness variables were shown to be superior. Comparisons were made to empty NPs (or comparable; *), free cargo (**), or other types of coated NVs (***) when this control was not taken into account experimentally.

####  Tumor microenvironment-targeted therapies

 This is another application of EM-NPs to managing cancer. Furthermore, in order to improve the therapeutic efficacy of EM-NPs for cancer, some authors created a nanoparticle that will only target tumour tissue. Such studies were carried out by According to research, surface functionalization of EM-NPs with targeting functionalities can be used to promote active medicine penetration into solid tumour tissues, boosting therapeutic efficacy.^60^ An example of such studies is Zhang and Chen, who used a lipid insertion technique to functionalize EM-NPs with an antibody for epidermal growth factor receptor, which is overexpressed in numerous human solid tumors, and the iRGD peptide to actively target colorectal or gastric cancer cells in their study. When compared to non-coated GA or PTX, these EM-NP methods with gambogic acid or PTX displayed significantly enhanced targeting, therapeutic efficacy, and biocompatibility in vivo.^61,62^ In comparison to non-coated nano erythrocytes or free DOX, further investigations using nano erythrocytes coated with DOX and functionalized with FA-PEG via the EDC/NHS reaction demonstrated enhanced drug accumulation in tumour cells and a clear anticancer effect in the H22 hepatocellular carcinoma in vivo model.^63^ Table 2 gives the list of ligands used for the surface modification of EM-NPs.

**Table 2:** Ligands used to modify the surface of EM-NP in order to develop better tumour microenvironment targeted therapies

**Modified ligand**	**Role**
Arg-Gly-Asp (RGD)	The uptake of tumor cells is increased.
Folic acid	Selective tumor cell identification; therapeutic efficacy and reduced toxicity
Mannose	Antigen-presenting cells in draining lymph nodes were targeted, and the metastatic load was reduced.
Dextran	Increased treatment effectiveness by targeting CD206 + cells
CDx peptides	Endothelial cells at the blood-brain barrier are being targeted for increased therapeutic efficacy.
Triphenylphosphonium	Mitochondrial targeting and increased O2 levels
Anti-epithelial cell adhesion marker	Improved uptake of cancer cells and therapeutic efficacy.

####  Functionalization of erythrocytes-based nanomedicine

 It is desirable to remove the barriers to tumour cell internalisation when using EM-NPs for the treatment of illnesses, particularly malignancies. Usually, this is done by either functionalizing RBC-NP to improve the liquid’s ability to permeate the tissue or by functionalizing the carrier with a ligand that binds the overexpressed tumour antigen to enable cancer targeting and reduce side effects. By employing the cell-impermeable linker NHS-PEG-maleimide, Zhou et al^39^ were able to maintain the recombinant hyaluronidase, PH20 (rHuPH20) molecules’ enzymatic activity while stabilising their attachment to the erythrocytes’ outer domain. The NPs were subsequently covered by the functionalized erythrocytes. With the same enzymatic activity, rHuPH20-conjugated EM-NPs in the gel doubled the free rHuPH20 diffusion effectiveness, but the EM-NPs alone barely diffused into the extracellular matrix-mimicking gels. When compared to RBC-NP with 10 U of free rHuPH20, conjugated rHuPH20 enhanced the amount internalised or attached to PC3 cells by three times. These findings indicated that the conjugated rHuPH20 might support NP transport more efficiently in ECM-imitating gels and the cytoplasmic HA matrix of PC3 cells. Furthermore, the EM-NP blood circulation time was unaffected by the rHuPH20 alteration. Thus, while including additional necessary functionalities, this functionalization technique maintains the underlying native cell membrane features.

 Targeting selection is a crucial technique to effectively avoid pharmacological side effects on healthy cells and tissues while using EM-NPs for the treatment of cancer.^64-66^ There are numerous current initiatives to increase the targeting effectiveness of this method, some of which involve chemically conjugating carboxyl, amine, or sulfhydryl groups on the surface of cell membranes.^67-69^ The original immunological escape function of RBCs will be lost as a result of these techniques, which cause chemical reactions and the inactivation of membrane proteins on the surface of RBC membranes. To functionalize RBC membranes, Fang et al^70^ devised a lipid insertion approach, using ligand-linker-lipid conjugates as targeting ligands. This study used folate, which has small molecules, and AS1411, an aptamer that targets nucleolin, which has larger molecules, as ligands to create targeting ligands. Flow cytometric and fluorescence imaging analysis revealed that model cancer cells took up modified erythrocyte membrane-coated NPs 8 and 2 times more than unmodified cells, demonstrating a strong targeting impact. The targeted ligand can access the cell membrane surface spontaneously with the help of lipid chains and the dynamic membrane bilayer structure, effectively protecting the membrane proteins from chemical reactions. Once the carrier’s absorption by the tumour cells is increased through targeting alteration, the entire release of the medicine becomes a serious problem. Utilizing light-sensitive nanocarriers or co-loading chemotherapeutic drugs and PS to accelerate drug release can be used in combination with chemotherapy to provide great anti-tumor effects.^35,46,71,72^ UCNPs are typically ligand-modified for cancer targeting, but in biological fluids, NPs form “protein coronas” that cover the ligands on the surface of the particles and lessen their targeting properties. According to a recent study by Rao et al,^73^ FA can be functionalized with UCNPs coated with erythrocyte membranes to efficiently prevent protein adsorption, which improves targeting effectiveness and in vivo tumour imaging. Additionally, “CDX peptides,” a brain-targeted delivery system that combines EM-NPs with special targeting moieties generated from neurotoxins, have been demonstrated to have potent brain targeting properties and drastically lower drug toxicity.^74^

 The most notable aspect of this study is its successful fusion of cancer-targeted therapy and natural immune escape, both of which are essential in the treatment of cancers. The next step may be to investigate the impact of trgeting ligands on EM-NPs in vivo and further highlight the promising cancer treatment prospects of this technique. In addition to the change in membrane surface (Table 2), the idea of merging two separate cell membrane sources to create novel bio-coatings has been put forth. They employed a hybrid membrane that combines platelet and erythrocyte membranes, which has been widely used in nanocoating to improve targeting in recent years, to coat the PLGA cores. The authors confirmed the successful transfer of the protein markers on the membrane surface following a series of in vitro and in vivo characterizations. They also showed that the RBC-platelet hybridization membrane enables the PLGA cores to function simultaneously with immune escape and targeting. This offers a clear direction for maintaining the unique characteristics of various cells through the fusion of other particular functional membranes, bypassing the constraints of the current multi-functional alterations of NPs. The method also creates a new area for the advancement of biomimetic NPs and enhances the applicability of emerging nano-carriers with complicated surface chemistry.^75^

####  Phototherapy 

 The inherent heterogeneity of a tumor cell makes it difficult to eradicate tumors with a single treatment. To combat this, one feasible alternative is to use a mix of numerous medicines with different mechanisms. Phototherapy is a laser-based treatment that uses optical absorption materials to transform energy from laser irradiation into heating, which is then used to kill tumour cells. This therapy has a high level of selectivity and can prevent injury to non-targeted areas.

 Piao et al were the first to investigate such research using erythrocyte membrane for the enhancement of photothermal therapy (PTT). When compared to non-functionalized nanocages, these biomimetic techniques improved circulation time and tumour uptake after irradiation. They discovered that RBC membrane-coated gold nanocages increased PTT efficacy, resulting in a faster decrease in tumour development and a 100% survival rate after 45 days, compared to bare nanocages or PBS-treated mice, which had an 80% or 20% survival rate, respectively.^71^

 In recent work, Rao et al effectively demonstrated the production of iron oxide magnetic NPs covered with erythrocyte membrane utilising a microfluidic electroporation approach. When compared to those made using traditional procedures, these EM-NPs had a full membrane coating and greater PTT therapeutic effectiveness.^35^

 According to this research, the EM-NPs obtained the photothermal conversion characteristics from their inner cores and the prolonged blood retention from the RBC membrane covering. In other investigations, RBC membrane-coated melanin NVs were established as a platform for in vivo antitumor PTT employing melanin as a photothermal agent. In A549 tumor-bearing mice, these EM-NPs had considerably better PTT efficacy than bare melanin NVs.^76^ These photothermal compounds can also be coupled with chemotherapeutic medications to increase their release, resulting in synergistic photothermal chemotherapy.

####  Combination therapies

 The NPs’ preserved erythrocyte membrane acts as a diffusion barrier, preventing rapid drug release into the bloodstream.^51^ This barrier, on the other hand, can result in a more effective release of drugs at the tumour location. To overcome this barrier, phototherapy can be used, where phototherapy can cause ablation of the RBC membrane and thereby promote the release of drug directly at the target site, giving high therapeutic efficacy and low side effects.

 In this approach, Wang and colleagues^77^ used erythrocytes and melanoma cells to disguise DOX-loaded hollow copper sulfide (CuS) NPs with a bioinspired covering for combination therapy (EM-NP-B16m@DCuS NPs). In comparison to noncoated CuS NPs, these NPs had a typical core-shell structure with a hollow core and a homogeneous outer RBC membrane shell. [EM-NP-B16]@CuS NPs were also larger than bare CuS NPs, and the zeta potential changed from 16 mV to 23 mV after CuS NPs were coated with RBC-B16 membrane, demonstrating that CuS NPs were shielded by the more negatively charged outer membrane surface. In vivo, the photothermal activity of CuS NPs coated on RBC membranes was validated. B16F10 tumor-bearing mice treated with [EM-NP-B16]@CuS NPs and [EM-NP-B16]@DCuS NPs showed a temperature increase of 52.6 °C and 51.2 °C, respectively, under NIR laser irradiation, but mice treated with normal saline only raised the temperature to 40.1 °C. Furthermore, with NIR laser irradiation, this biomimetic method exhibited a significant combinatorial chemo-PTT with a 100% melanoma tumour growth suppression rate, whereas free DOX and empty [EM-NP B16] @ CuSNPs showed only a 10% and 86% inhibition rate, respectively. There was no evidence of toxicity^77^. These findings have a lot of promise for photothermal ablation of tumour tissues and hyperthermia-responsive medication release. Su et al showed a combined therapy using erythrocyte membrane-mimetic NPs in combination with NIR laser-responsiveness, where NIR activates cellular uptake and releases the drug in a controlled fashion. A NIR dye was implanted in RBC membrane shells, and a hybrid polymeric nanoparticle core loaded with PTX thermoresponsive lipid was used to construct the hybrid polymeric nanoparticle core. When compared to free dye, the dye fluorescence in the NPs can be exploited for in vivo tumour imaging, boosting the circulating half-time by 12.3 times. Under the effect of NIR laser irradiation, tumour uptake of NPs was 2.1-fold higher than without irradiation. The light-induced hyperthermia can disrupt the erythrocyte-mimetic NP structure, resulting in rapid PTX release. In vivo, our method delivered a synergistic chemophotothermal treatment, reducing over 98% of lung metastasis and lowering breast tumour development by 3.6-fold compared to empty NPs.^78^

 Pei et al recently suggested a new EM-NPs method in which the inner core is mostly made up of PTX dimers employing tioketal (PTX2-TK) in combination with tetraphenyl chlorin (TPC), which is reactive to reactive oxygen species (ROS) and a photosensitizer. TPC-generated ROS will cause the PTX2-TK bond to be cleaved and PTX to be released under the right conditions of light irradiation. When compared to non-coated NPs, RBC membrane-coated NPs have a longer blood circulation period and better tumour accumulation—almost 4.6 times greater than in vivo investigations.^79^ DTX and IR780 iodide were co-loaded in erythrocyte membrane-coated PCL-based NPs (IR780/DTX-PCEC@EM-NPs) by Yang et al compared to non-coated NPs, these erythrocyte-coated NPs showed good stability and increased circulation time by about 2.12 times. Furthermore, although non-coated NPs decreased MCF-7 tumour development by 21.8%, RBC-coated NPs inhibited tumour growth by 45.1%, indicating their potential for future use as an imaging-guided chemo-PTT for breast cancer.^80^

 In other experiments, erythrocyte membrane-coated bovine serum albumin (BSA) NPs loaded with indocyanine green (ICG) and GA showed significant long-term circulation and avoided early drug leakage. These biomimetic structures dramatically suppressed tumour growth in HeLa tumor-bearing animals, inhibiting it by 87%, whereas free GA or NIR irradiation only inhibited tumour growth by 10% or 3%, respectively. These findings suggest that chemo-photothermal combination therapy can improve the therapeutic potential of single therapies in vivo.^81^

 Aside from chemo-phototherapy combinations, different treatment combinations like radiation and EM-NPs have also been studied.^82,83^

 Because oxygen is required to produce radiation-induced cell destruction, traditional radiotherapy suffers from a serious problem where low amounts of oxygen (hypoxia) at the tumour site restrict its therapeutic effectiveness.^84^ Perfluorocarbons (PFCs) are inert compounds with a high oxygen solubility that can be employed to give artificial oxygen to tumour hypoxia sites to overcome this problem. In one study, Gao et al integrated PFC into PLGA-NPs (polylactic glycolic acid NPs) covered with RBC membrane. These erythrocyte-coated PFC NPs have demonstrated a high oxygen loading efficiency as well as lengthy blood circulation periods. After IV injection, this method was proven to successfully supply O2 at the tumour location, alleviating tumour hypoxia and thereby enhancing radiation efficiency.^82^

 The hypoxic tumour microenvironment (TME) can be a problem because photodynamic therapy is an oxygen-dependent treatment. In this context, Wang et al created ROS-sensitive bioinspired NPs co-encapsulated with the photosensitizer Ce6 (chlorin E6) and a hypoxia-activated prodrug tirapazamine (TPZp). RBC membrane and RGD peptide were also used to reform these NPs. Ce6 for PDT created ROS in response to light irradiation, causing the ROS-responsive NPs to dissociate. The activation of the TPZ by the local hypoxic TME improved the therapeutic effect much more. As a result, by integrating the synergistic effects of tumor-targeted PDT with hypoxia-activated chemotherapy, these biomimetic NPs greatly increased anticancer efficacy.^83^ Researchers used RBC and 4T1 tumour cell membrane camouflage NPs co-loaded with photosensitizers ICG and tirapazamine (TPZp) to combine PDT and chemotherapy. In comparison to noncoated nanocarriers, this treatment combination reduced tumour progression in vivo by 1.9 times.^85^

 Zhang et al have created RBC membrane shrouded metal-organic framework (MOF) NPs loaded with glucose oxidase (GOx) and TPZp for starvation-activated cancer therapy. These NPs effectively gathered inside the tumour cell, causing hypoxia as a result of GOx-induced hunger, which triggered TPZp activation. As a result, combining hypoxia-activated chemotherapy with fasting therapy resulted in a synergistic slowing of tumour development.^86^ Others integrated GOx and Mn2 (CO) 10 (carbon monoxide (CO) donor) in erythrocyte membrane-coated PLGA NPs to boost in situ CO generation for combined cancer cell energy starvation and gas treatment. Energy deprivation and CO gas production inhibited tumour cell proliferation in vitro, resulting in mitochondrial malfunction, and this arrangement showed augmentative synergistic efficacy in inhibiting breast tumour growth.^87^ Table 3 summarizes all the EM-NPs for combination therapy in cancer.

**Table 3:** EM-NPs for combination therapy in cancer

**EM-NVs composition**	**Drug and adjuvant used**	**Combination name**	**Benefits of Combination.**	**Therapeutic** **efficacy**	**Experimental model**	**Ref**
CuS@EM-NP-B16m	Doxorubicin	DDR + PTT	Circulation, targeting ability, and treatment efficacy have all improved.	1.2-Fold* 10-Fold**	Murine B16F10 melanoma	^77^
PCL-DPPC@EM-NP-DIR	Paclitaxel	PTX + PTT	Improved tumor cell uptake and chemo-photothermal treatment that functions together	3.6-Fold* 5.6-Fold**	Murine 4 T1 breast tumor	^78^
PEG-b-PDLLA@EM-NP	tetraphenyl chlorin + Paclitaxel - Tioketal	DDR + PDT	Longer blood circulation, more medication loading, less toxicity, and better treatment efficacy	~10-Fold**	HeLa cervical tumor xenograft	^79^
ε-CL + PEG (PCEC)@EM-NP	Docetaxel + IR780 iodide	DDR + PTT/ PDT + FI	Stability, circulation time, and antitumor activity have been improved.	~2.1-Fold	MCF-7 breast tumor xenograft	^80^
BSA@ EM-NP	Indocyanine green + Gambogic acid	DDR + PTT	Loading efficiency and therapeutic efficacy are both improved.	~8-Fold**	HeLa cervical tumor xenograft	^81^
PLGA@ EM-NP	Perfluorocarbon	O2 + RT	Enhanced blood circulation, extracellular diffusion, and therapeutic benefit due to increased oxygen content	~3-Fold** ~9-Fold**	Murine 4 T1 breast tumor	^82^
mPEG-b-PBBA@ EM-NP -iRGD	Tirapazamine + Chlorin e6	DDR + PDT	Selective tumor accumulation, enhanced tumor penetration, and therapeutic efficacy when used together	~4-Fold***	Murine 4 T1 breast tumor	^83^
MSN@ EM-NP -4T1m	Tirapazamine + Indocyanine green	DDR + PDT	Increased treatment effectiveness and reduced macrophage clearance	~1.9-Fold	Murine 4 T1 breast tumor	^85^
ZIF-8@RBCm	Tirapazamine + Glucose oxidase	DDR + ST	Higher tumor accumulation, effective GOx-depletion treatment, and synergistic pharmacological efficacy	2-Fold***	Murine CT26 CRC tumor	^86^
PLGA@ EM-NP	Glucose oxidase + Carbon Monoxide-donor	ST + COT	CO gas therapy with energy deprivation has shown good synergistic therapeutic efficacy.	~2.7-Fold	Murine 4 T1 breast tumor	^87^

CO gas therapy (COT); Drug delivery (DDR); Fluorescence imaging (FI); Photodynamic therapy (PDT); Photothermal therapy (PTT); Radiotherapy (RT); Starvation (ST); Poly(ethylene glycol)-block-poly(D, L-lactide) (PEG-b-PDLLA); Mesoporous Silica NPs (MSN); Zeolitic imidazolate framework-8 (ZIF-8). Comparisons were made to empty NVs (or equivalent; *), free cargo or single therapy (**), or other types of coated NVs (***) when this control was not taken into account experimentally.

 The conclusion drawn from this section of the review is that this combination therapy using EM-NP could offer a viable alternative to conventional therapy, with benefits such as fewer side effects compared to standard treatment, slower development of drug resistance, a lower rate of treatment failure, and, most importantly, lowering the financial burden associated with the development of new drugs by providing the most cost-effective approach.

####  Imaging and diagnosis

 Imaging and diagnosis methods serve a crucial role in the early diagnosis of many cancers; they also aid in determining the stage at which cancer has progressed, as well as the precise location of the tumor, which aids in determining the best course of action if surgery or other treatments are required, or in preventing cancer relapse. EM-NPs have been studied extensively to improve the diagnostic quality of molecular imaging techniques such as fluorescence imaging, magnetic resonance imaging (MRI), photoacoustic imaging (PAI), and positron emission tomography (PET). Upconversion NPs (UCNPs), for example, have been investigated for in vivo cancer imaging because they have good chemical and optical characteristics and can convert light from the NIR to the visible range. In this regard, Rao and colleagues developed UCNPs with RBC membranes functionalized with folic acid, and the resulting bioinspired FA-EM-UNCNPs, when exposed to human plasma, prevented the formation of the protein corona. FA-EM-UNCNPs showed the strongest green upconversion luminescence at the tumour location both ex vivo and in vivo when evaluated under NIR irradiation, suggesting their efficient targeting capacity for MCF-7 breast tumour xenografts.^73^ Sindhwani et al recently camouflaged a folic acid tumor targeting UCNPs (FA-EM-UCNPs) with RBC membranes to make them undetectable to the immune response and clearance by the host system. When intravenously injected into 4T1-tumor-bearing mice, fluorescence microscopy of UNNP revealed that FA-EM-UCNPs displayed rapid accumulation, long-term retention, and decreased immune system uptake. In terms of the possibility of employing these bioinspired NPs in MRI and PET imaging for tumour detection in vivo, the FA-EM-UCNPs dramatically improved the MRI signal, indicating increased NP circulation time at the tumour site. A combination of pre-targeting and in vivo click chemistry was also used to successfully perform PET imaging of the EM-UCNPs using short half-life radionuclides.^88^

 Because of its deep tissue penetration and fine spatial resolution, PAI is also one of the most essential imaging modalities, with promising implications for clinical cancer diagnosis. However, PAI techniques are less commonly used due to their insufficient tumour catalytic response, which led to the creation of this technology. In 2018, Ding et al developed an exosome-like nanozyme particle coated with an RBC membrane functionalized with folic acid for H2O2-responsive PAI of nasopharyngeal cancer in vivo. This method accumulated efficiently in tumours and selectively triggered catalytic PAI, implying that an exosome-like nanozyme vesicle is an appropriate nano-strategy for creating deep-tissue tumor-targeted catalytic PAI in vivo.^89^ Yang et al encapsulated IR780 iodide and DOX in erythrocyte membrane-coated polycaprolactone-based NPs and demonstrated that these EM-NPs can be used not only for diagnosis as a FI/PAI dual-model imaging probe but also for tumour treatment via phototherapy and chemotherapy in one study. As a result, these EM-NPs can serve as a viable model for future FI/PAI-guided photochemotherapy therapies for breast cancer.^80^

 The detection of malignant circulating tumour cells (CTCs) is another novel application of EM-NPs. CTCs are cancer cells that escape from the original tumour and enter the bloodstream, where they move to various organs. These CTCs cells are primarily involved in the cancer’s metastatic phase and may have predictive relevance in cancer diagnosis, prognosis, and treatment selection.^90^ However, due to a large number of leukocyte impurities in the sorting process, current CTC detection methods are still limited. As a result, there is a pressing need for novel ways to increase the quality of retrieved samples. Several scientists presented techniques for erythrocyte engineering,^62^ a and RBC membrane biomimetic coating has recently gained interest for this purpose. Chen et al^62^ used chemical crosslinking and hydrophobic interaction, respectively, to coat folic acid and MNs on the surface of RBCs. When this designed EM-NP is fed to CTC cells, it quickly adheres to them, forming CTC-erythrocyte conjugates, which were subsequently isolated in a magnetic field. When CTC-erythrocyte conjugates are treated with RBC lysing buffer, the conjugation breaks down, and CTCs are retrieved by centrifugation. This CTC-erythrocyte conjugates method captures CTCs with high purity ( > 75%) and efficiency. Following that, in vitro experiments could be used to re-culture and multiply these cells.^62^ Meng and colleagues have camouflaged immunomagnetistic NPs with erythrocyte-derived vesicles, which do not absorb any biomolecules but preserve CTC targeting when exposed to plasma. This method had a cell isolation effectiveness of 95.7% in spiked blood samples, compared to 60.2% for non-coated immunomagnetistic NPs, and was able to effectively separate CTCs in 28 of 30 prostate cancer patient blood samples.^92^ Aside from that, Chen et al created nanovesicles from RBC membranes that had been surface-modified with FA and fluorescein Cy5. For CTC capture and tumour imaging, our method revealed remarkable tumour targeting capabilities, including both in vitro and in vivo. Furthermore, these RBC nanovesicles avoided nonspecific protein adsorption, achieving excellent CTC capture purity of 90% in whole blood, compared to 30% for standard immunomagnetic beads.^91^

**Figure 5:**
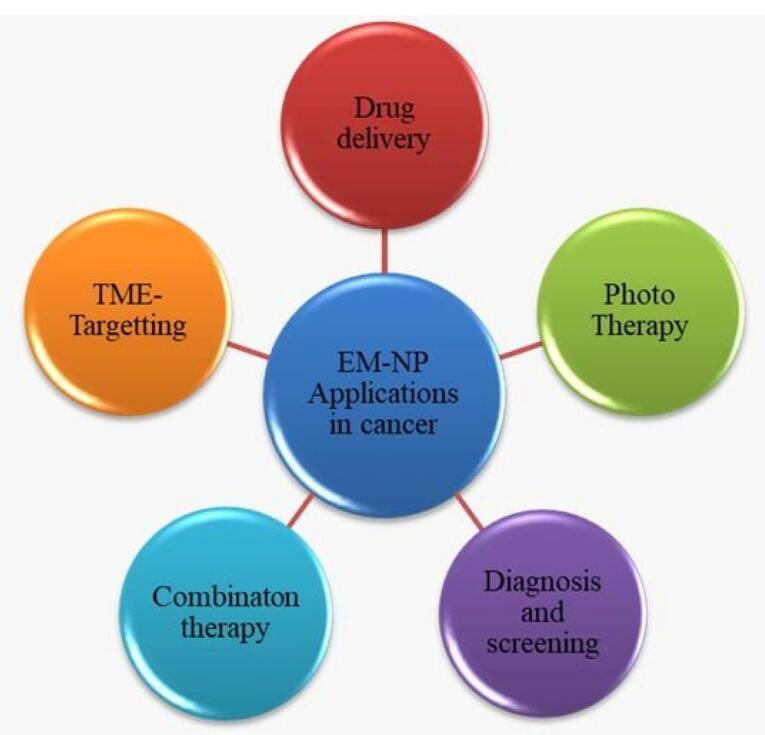
Applications of Erythrocytes mimicking nanoparticles in treating cancer.

 Overall, EM-NPs could be a helpful choice not only for improving traditional cancer therapy but also for imaging approaches, providing critical support in cancer diagnosis and progression evaluation. A summary of application erythrocytes NPs has been given in Figure 5.

## Toxicity and Immunogenicity of Erythrocytes based nanoparticles

 The “self” nature of RBCs in the biological milieu, have made RBC-based drug delivery systems gain attention with an emphasis on avoiding phagocytic clearance, resulting in prolonged circulation duration and less toxicity. Researchers examined the RBC membrane-derived vesicle circulation properties in comparison to synthetic materials and concluded that CD47 retention is the primary factor preventing RES absorption and extending plasma circulation time.^93^ In animal experiments, RBC-derived constructions are biocompatible and possibly non-toxic. In mice, where just 20% of the injected particles could be found in the hepatic circulation after 48 hours, they did not exhibit any long-term toxicity.^94^ Through the analysis of histology samples and serum biochemistry profiles, biotoxicity has been assessed in vivo. It is crucial to conduct more short- and long-term investigations to prove that these constructions behave in a non-toxic way.^35^ RBCs are naturally biocompatible, biodegradable, and appear to be immunological-neutral; nevertheless, additional research is required to rule out any other immune toxicity, complement activation-related pseudo-allergy, or hypersensitive states. It is important to understand how the complement system interacts with vesicles produced from RBC membranes. Before human administration, researchers advise assessing the complement activation-related pseudo-allergy and hypersensitivity potential of RBC-based delivery systems. In terms of confirming therapeutic potential and immunogenicity, using components in RBC-based delivery systems that have received clinical approval may be beneficial. The evaluation of immunological compatibility and systemic toxicity also depends heavily on the choice of appropriate in vivo animal models.^95^

 It is safe to say that RBC-based NPs are non-immunogenic and largely safe for usage, but the researcher also noted that more thorough short- and long-term toxicity studies are needed to support the safety and non-immunogenicity of the erythrocyte-based NPs. Since there is currently a dearth of information on the toxicity of erythrocyte-based NPs, claiming that they are safe is unfair, researchers have recommended doing extensive toxicity and immunogenicity studies.

## Challenges that are faced in the translation of EM-NP for clinical use

 The concept of modifying the surface of the nanoparticle with an erythrocyte membrane and using it for the treatment of various cancers was found to be very successful when the results of different preclinical studies were reviewed. Erythrocytes providing their natural membrane to the surface of a nanoparticle gave an added advantage to this strategy, which is easy immune evasion, prolonged blood circulation, easy tumour penetration, easy targeting of the nanoparticle to the tumour site, increased therapeutic efficacy, and many other advantages Despite these numerous benefits The EM-NP drug delivery system is confronted with some challenges that make clinical translation difficult and prevent it from reaching its full potential. For a delivery system to achieve clinical translation, it should not only show adequate in vivo activity but also show long-term stability and be easy to scale up. There should be no batch-to-batch variation, and there should be effective encapsulation efficiency. There are no long-term toxicity studies available related to this delivery system that would provide an opportunity to do so. Because this strategy is based on the biological membrane, it faces numerous challenges such as membrane extraction, purification of the RBC membrane, and storage issues. Additionally, the RBC membrane contains surface proteins that, if denatured, will result in an immediate immune response and other side effects.

## Conclusion

 Despite the numerous challenges, EM-NPs can still be regarded as a unique delivery technology because they have demonstrated themselves in preclinical models for a wide variety of clinical and diagnostic potential uses for cancer. With a wide range of biomedical applications, including drug administration, photodynamic therapy, combination therapy, and diagnosis, the advancements in EM-NP techniques for cancer are undeniably impressive. Additionally, because of their potential to deliver medications of interest at much lower concentrations to targeted locations with little adverse effects, these biomimetic nanovehicles can be used as a “neo” adjuvant to present therapies. Despite significant advancements, EM-NVs still require a great deal of fine-tuning to ensure a proper clinical translation. Exploiting different manufacturing processes that can achieve higher membrane extraction and encapsulation efficiency, as well as ensuring the development of optimal methods that reduce batch-to-batch variation and the potential to scale up, should most likely be pursued to improve the potential to translate the nanosystem to clinical practice. These are the most critical issues that must be addressed to get EM-NPs from the lab to the clinic. To conclude, EM-NPs are still young nanosystems that are being enthusiastically improved to ease clinical application as soon as possible.

## Competing Interests

 Authors declares no conflict of interests.

## Ethical Approval

 Non-applicable.
